# Pseudo-Renal Failure in the Context of Traumatic Bladder Rupture

**DOI:** 10.7759/cureus.32360

**Published:** 2022-12-09

**Authors:** Matthew Newman, Nityanandan G, Niamh Smyth

**Affiliations:** 1 Paediatric Surgery, Aberdeen Royal Infirmary, Aberdeen, GBR; 2 Urology, Aberdeen Royal Infirmary, Aberdeen, GBR; 3 Urology, NHS Forth Valley, Scotland, GBR

**Keywords:** aki mortality, acute kidney injury, bladder rupture, trauma, urology

## Abstract

The bladder is both an intraperitoneal and extraperitoneal structure. Its anatomical position increases its risk of rupture. The resultant urine leak or extravasation can be intraperitoneal, extraperitoneal, or even both-with the former leading to more sinister outcomes. Intraperitoneal bladder rupture can lead to urinary ascites which along with anuria and abdominal pain, can present with an apparent abrupt decline in renal function as the creatinine-rich products diffuse across the peritoneal membrane.

Glomerular filtration rate, a measure of kidney function is related to the levels of serum creatinine. Clinicians can therefore misdiagnose their patient with acute kidney injury when the serum creatinine is elevated as a consequence of urine being present in the peritoneal space.

This is a case report of a 62-year-old male with pseudo-renal failure following intraperitoneal bladder rupture after a fall face-forwards three hours previously. The fall was due to icy conditions outside and no preceding symptoms were reported. He presented to the Accident and Emergency department with abdominal pain and no other positive symptoms.

The patient had a good World Health Organisation (WHO) performance status with a background of hypertension, diabetes, and hypercholesterolemia. The bedside examination of the patient revealed a distended, abdomen with peritonitis. There were no signs of urogenital trauma. Blood testing revealed a low estimated glomerular filtration rate (eGFR) and raised creatinine (eGFR of 7 millilitres/minute and creatinine of 658 micromoles/litre).

Computerised tomography examination of the abdomen and pelvis (CTAP) revealed free fluid within the peritoneal cavity and an irregular bladder wall. A CT cystogram and consultation with urology led to the diagnosis of intraperitoneal bladder rupture.

The patient’s renal function from an initial set of blood tests was reduced. This was not a true impairment in renal function but rather a complication secondary to extravasation of urine in the intraperitoneal space, ie., pseudo renal failure. This supposed impairment in renal function had numerous implications. It affected the choice of antibiotics; amoxicillin and gentamicin were given at a reduced dose due to the patient’s renal function and the patient was prepared for operation theatre.

The patient's blood creatinine was falsely elevated at 658 micromoles/litre due to the diffusion of creatinine from the free urine in the peritoneal space into the blood. This painted a false image of renal failure and protracted the clinical decision-making process. Relatively simple measures like an ascitic tap could have helped to differentiate this from a true acute kidney injury and could have resulted in quicker and more effective treatment of this patient.

The patient went on to have bladder repair under urology. His follow-up cystogram four weeks post-operation did not show any leak.

## Introduction

Bladder rupture has a high mortality rate if not treated quickly and effectively. Due to urinary ascites and auto-dialysis, clinicians can misdiagnose acute kidney injury leading to poor patient management in acute settings. The message we would like to convey in this case report is for the clinician to consider the effects of urinary ascites on biochemical investigations, and to raise awareness of the prospect of pseudo-renal failure in the context of traumatic bladder rupture.

## Case presentation

Initial presentation

A 62-year-old Caucasian male self-presented to the Accident and Emergency department with abdominal pain following a fall face-forwards three hours earlier. The fall was due to icy conditions outside and no preceding symptoms were reported. The abdominal pain was described as generalised, severe, constant and without radiation. He had no associated bowel symptoms or episodes of vomiting. Prior to the incident, he reported the need to pass urine, however, this urge had subsided immediately after the event without micturition.

He was independent in activities of daily living and would walk approximately 2 miles a day. He had a past medical history of hypertension and hypercholesterolaemia for which he took an angiotensin-converting enzyme inhibitor and a statin. He had a BMI of 35.9. He reported that he suffered from type 2 diabetes mellitus which was diet controlled. Upon review of previous haematology results, however, there had been an HbA1C of 67 mmol/mol recorded approximately 18 months prior to this presentation. This did not appear to have been acted upon. 

An examination in the emergency department revealed a firm, distended abdomen with peritonism in the lower half. There was no blood or other evidence of trauma present at the urethral meatus and his testicle examination was unremarkable. The patient was apyrexial, had a heart rate of 120 bpm, blood pressure of 111/84 mmHg, respiratory rate of 30 per minute and oxygen saturations of 96% on 2L O2 via nasal cannula.

Investigations

A focused assessment with sonography in trauma (FAST) at the emergency department showed free fluid within the pelvis.

**Figure 1:**
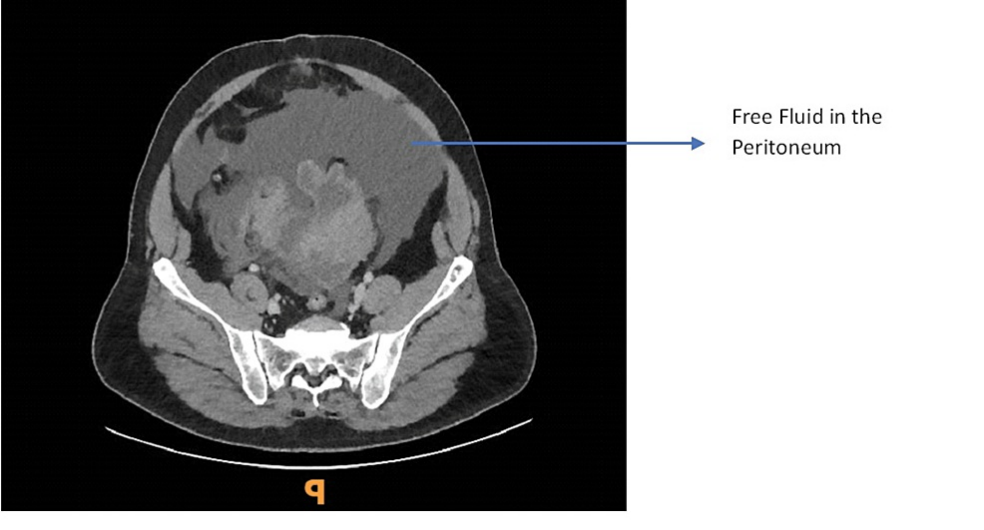
Free Fluid in the Peritoneum

Laboratory data revealed severe renal dysfunction with hyponatremia, uraemia, hyperglycemia and a raised lactate. A contrast computerised tomography examination of the abdomen and pelvis (CTAP) was reported to show a large volume of free fluid within the peritoneal cavity (Figure 1). There was a large irregular bladder mass with perivesical soft tissue extending over the left side and irregular contour of the bladder wall/roof. A delayed urographic phase non-contrast CT covering the pelvis was performed 15 minutes after the initial contrast CTAP, which showed contrast opacification of the bladder lumen though no contrast extravasation was identified (Figure 1). The on-call radiologist suggested a conventional CT cystogram via a transurethral catheter, however, the urology team felt confident enough with examination findings and CT suspicion to make a diagnosis of intraperitoneal bladder rupture.

Treatment

Amoxicillin and gentamicin were given at a reduced dose, in accordance with local hospital policy and renal prescribing guidelines, due to the patient’s renal function and the patient was prepared for the operation theatre.

The urology team performed an emergency laparotomy during which approximately three litres of fluid were suctioned from the abdomen. An enlarged bladder was found with a 5 cm defect in the dome. There was also a sizable intravesical clot and mural haematoma which would account for the irregular mass seen on CT. The team performed a two-layer closure of the defect with 1-0 Vicryl. To ensure full closure of the injury and no secondary defect, the bladder was filled with 0.9% saline intraoperatively.

The patient was transferred to the ICU for a short post-operative stay before being stepped down to a ward. His creatinine seven hours post-operation was 340 micromoles/litre (normal value 61.9 to 114.9 micromoles/litre) [1] and after 30 hours post-operation was 129 umol/L. A repeat HbA1C the morning after surgery was 86 mmol/mol (normal value < 48 mmol/mol) [1]. Rectal examination revealed an enlarged smooth prostate. His renal function had normalised 36 hours post-operation and he was discharged home on day six with a catheter in situ and diabetes follow-up.

Outcome and follow-up

**Figure 2:**
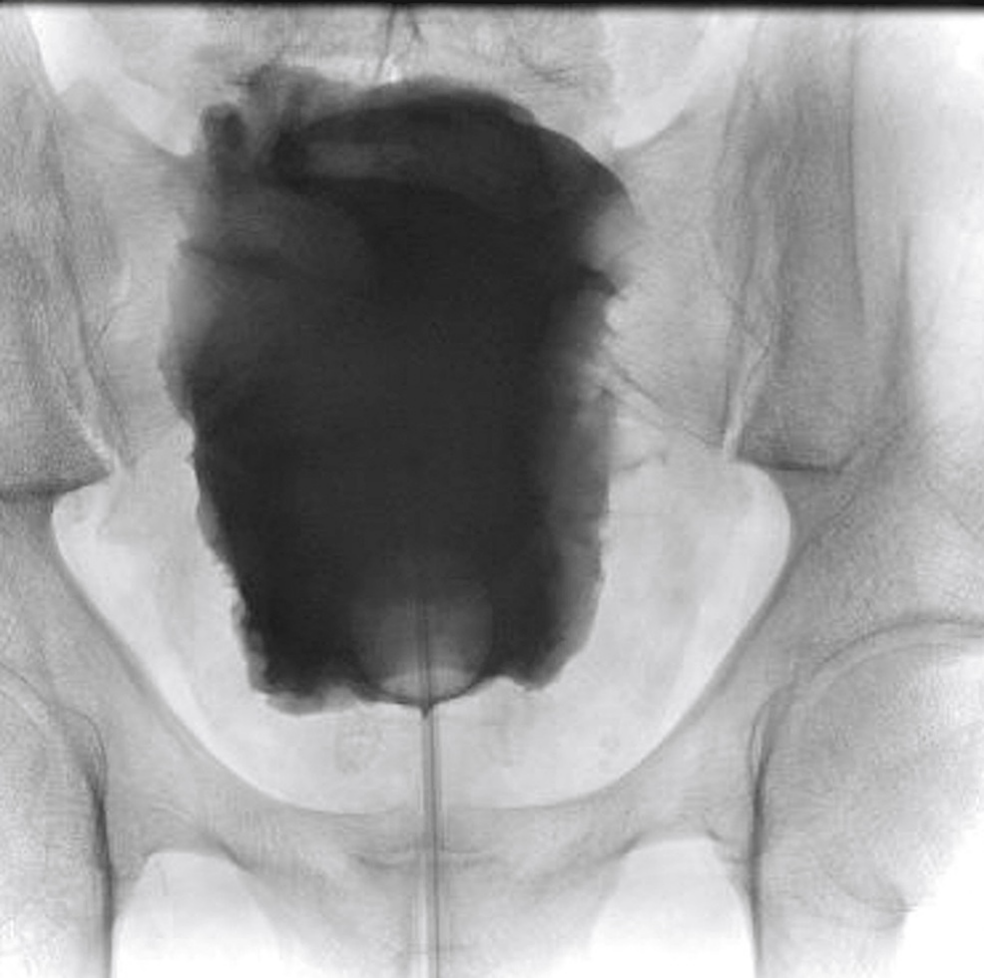
Retrograde cystogram showing no contrast leak from the bladder

Four weeks after discharge, he was followed up with a retrograde cystogram (Figure 2) and underwent a trial without a catheter. The retrograde cystogram showed no leak (Figure 2) but the patient failed his trial without a catheter.

We brought this gentleman back for review in the clinic three months later for a further trial without a catheter, however, once again, this failed. One year on, the patient is using intermittent catheterisation to help with voiding and is undergoing physiotherapy to improve bladder function.

## Discussion

An intraperitoneal bladder rupture can cause urinary ascites resulting in creatinine-rich fluid within the abdominal cavity. The higher concentration of urinary waste products within the peritoneum compared with the blood creates a concentration gradient encouraging the reabsorption of azotemic products back into the bloodstream, therefore resulting in the findings of electrolyte abnormalities (hyponatraemia and hyperkalaemia) and renal dysfunction (raised creatinine and an apparent reduction in estimated glomerular filtration rate (eGFR)) [1]. The abnormalities seen on biochemical analysis do not represent deterioration in renal function but are in fact due to reverse dialysis across the peritoneal membrane. It can provide a misleading picture of the patient’s clinical state and leads to less-than-optimal care for the patient such as an under-dosing of antibiotics.

Bladder rupture is a relatively rare complication of blunt abdominal trauma representing 0.36% of patients admitted with blunt abdominal trauma in a study of over 15,000 patients. Rupture can be either extraperitoneal or intraperitoneal, depending on the mechanism of injury. Extraperitoneal ruptures will tend to be associated with pelvic fractures, whereas intraperitoneal ruptures will be more likely associated with blunt abdominal trauma [2]. Specifically, with intraperitoneal rupture, the dome of the bladder is most at risk of rupture due to its weakened area around the urachal attachment [3]. Although bladder rupture is rare in trauma, its mortality can range from 10-22% [3] and therefore it is an important injury to consider. For patients who suffer an extraperitoneal bladder rupture, these can usually be managed conservatively with catheter drainage and prophylactic antibiotics. Healing usually takes around three weeks, however, those with an intraperitoneal rupture should be managed surgically with a laparotomy and surgical repair [4].

Pseudo-renal failure in the context of urinary bladder rupture as described here is rare [5-8]. In addition to abdominal trauma, pseudo-renal failure has been reported in the literature due to bladder perforation following laparoscopic surgery [1], bladder diverticulum perforation [7], bladder infection [9,10] and neoplasmic invasion [11]. Spontaneous perforation has also been associated with radiotherapy for bladder tumours [12].

Focus assessment sonography in trauma can provide a rapid bedside screening tool to identify free fluid within the abdominal cavity, however, it is not reliable in distinguishing the nature of this fluid [13]. CT cystography could be performed and has proven to be highly specific in diagnosing bladder rupture by identifying extravasation of catheter-introduced contrast [14]. However, in situations where they may be a severe lack of time, the history, clinical examination findings of peritonism and standard contrast CT are sufficient to diagnose an intraperitoneal bladder injury.

It should be noted that an ascites: serum creatinine ratio greater than one is suggestive of an intraperitoneal urinary leak, therefore urinary ascites could be diagnosed through biochemical analysis of an ascitic tap. This may however not be warranted in the emergency department.

During the laparotomy, our patient was found to have an enlarged bladder. Three litres of urinary ascites were drained intra-operatively. It is therefore likely that our patient had some level of undiagnosed chronic urinary retention and had a distended bladder prior to his fall. A distended bladder is at far higher risk of traumatic rupture due to its projection into the abdominal cavity. Bladder distention caused by chronic urinary retention can occur for several reasons, most commonly due to benign prostatic hyperplasia (BPH). Our patient did not have a prior diagnosis of BPH, however on examination post-operatively he was found to have a smooth and enlarged prostate. Poorly controlled diabetes (which was demonstrated in this case by the elevated HbA1C) is a further risk factor for chronic urinary retention, thought to arise from impaired sensation from bladder distension removing cues for voiding.

## Conclusions

Bladder rupture can be classified as intra- or extraperitoneal. Intraperitoneal almost always requires operative management, whereas extraperitoneal can often be managed conservatively. Intraperitoneal bladder rupture can lead to creatinine-rich fluid within the peritoneal cavity. Therefore, eGFR may not represent true GFR and can have a significant impact on clinical decisions for investigations and treatment. eGFR corrects rapidly after bladder repair and peritoneal washout.
